# Incidence of major health events across metropolitan and regional areas: A 10+ year prospective study of 16,697 older Australians

**DOI:** 10.1111/ajag.70036

**Published:** 2025-05-07

**Authors:** Shay Farbotko, Alice Owen, Suzanne G. Orchard, Robyn L. Woods, Mark Nelson, Nigel P. Stocks, Andrew Tonkin, Rory Wolfe, John McNeil, Peter Gibbs, John Zalcberg, Joanne Ryan

**Affiliations:** ^1^ School of Public Health and Preventive Medicine Monash University Melbourne Victoria Australia; ^2^ Menzies Institute for Medical Research University of Tasmania Hobart Tasmania Australia; ^3^ Discipline of General Practice, Adelaide Medical School University of Adelaide Adelaide South Australia Australia; ^4^ The Walter & Eliza Hall Institute of Medical Research Melbourne Victoria Australia; ^5^ Department of Medical Oncology Peter MacCallum Cancer Centre Melbourne Victoria Australia

**Keywords:** chronic disease, dementia, independent living, risk factors, rural population

## Abstract

**Objective:**

To examine the prevalence of health risk factors by rurality status and the association of rurality and incidence of disability‐free survival (DFS), its components (death, dementia and physical disability), cardiovascular disease (CVD), cancer and underlying cause of death.

**Methods:**

Data came from the ASPirin in Reducing Events in the Elderly (ASPREE) trial and observational extension, ASPREE‐XT. Community‐dwelling Australians aged 70 years or older, with no prior CVD events, dementia or independence‐limiting physical disability, were recruited through General Practitioners between 2010 and 2014. Area of residence was classified as major cities, inner regional or outer regional/remote. Major incident health events were adjudicated by expert panels.

**Results:**

Participants (*n* = 16,697, median age 74 years; 55% female) were followed over a median 8.3 years. A small, but statistically significant higher prevalence of many health risk factors was found for individuals living outside metropolitan areas. Rurality was not associated with the incidence of DFS, dementia, physical disability or CVD events in adjusted Cox proportional hazards regression models. Compared to major cities, individuals in outer regions/remote areas had an increased risk of all‐cause death (HR: 1.17; 95% CI 1.02, 1.34) which appeared to be driven by fatal CVD (HR: 1.40; 95% CI 1.02, 1.83), while those in inner regions had a lower cancer incidence (HR: .89; 95% CI .82, .98).

**Conclusions:**

Incidence of DFS, dementia and physical disability did not differ according to rurality. Heightened risk of mortality was evident outside urban areas, possibly reflecting inequitable health service and access. Lower cancer incidence in inner regions requires further investigation.

**Policy impact**

The prevalence of key risk factors contributing to poorer later‐life health, and the incidence of all‐cause mortality and cancer, even after accounting for these, varied by rurality status. The development of place‐based strategies targeting modifiable conditions and health service access and education are needed to support equitable ‘ageing in place’.

## 1 INTRODUCTION

Prolonging years lived independently and in good cognitive and physical health is a crucial societal goal in the context of population ageing.^1^ However, despite increased life expectancy, there has been little improvement in healthspan.^1^, ^2^ Leading causes of mortality and functional disability in older age include dementia, cardiovascular diseases (CVD) and cancer.^2^, ^3^ Behavioural factors (e.g. smoking, excess alcohol use and physical inactivity) and unmanaged health conditions (e.g. depression, obesity, diabetes, high blood pressure and dyslipidaemia) are key contributors to increased risk of developing these diseases.^4^, ^5^ The prevalence of these health risk factors may vary by rurality, determined by a complex interaction of socio‐economic, environmental and structural influences.^5^

Older adults are typically overrepresented in more rural areas; hence, the importance of determining how rurality contributes to age‐related conditions and functional dependence.^6^, ^7^ There is some evidence that the risk of dementia^7^, ^8^, ^9^, ^10^ and persistent physical disability^11^ may be heightened for individuals living in more rural and remote regions. However, there is limited comparative data from large‐scale longitudinal studies in older adults to support this.^8^, ^12^ A better understanding of whether these health outcomes, and other key contributors to death and functional decline such as CVD and cancer, differ by rurality status will facilitate the development of place‐based preventive strategies and targeted policy aimed at healthy ageing equity.

The ASPirin in Reducing Events in the Elderly (ASPREE) longitudinal study provided a unique opportunity to analyse these gaps in knowledge by leveraging a large, geographically diverse community‐based cohort of older adults, with regular assessment and standardised adjudication processes. The aim of this analysis was twofold: first to examine differences in prevalence of modifiable health risk factors by rurality; and second to assess adjusted associations of rurality status with disability‐free survival (DFS), and its components of death, incident dementia and persistent physical disability, as well as CVD, cancer and cause‐specific mortality.

## 2 METHODS

### 2.1 Study design and participants

Australian data were from the ASPREE clinical trial of low‐dose aspirin and the observational extension (ASPREE‐XT).^13^ Participants were 16,703 community‐dwelling adults aged 70 years or older, with no history of CVD events and free of dementia, independence‐limiting physical disability and other major health concerns likely to be fatal within 5 years, recruited through General Practitioners between March 2010 and December 2014.^14^

### 2.2 Determinant: rurality status

**FIGURE 1:**
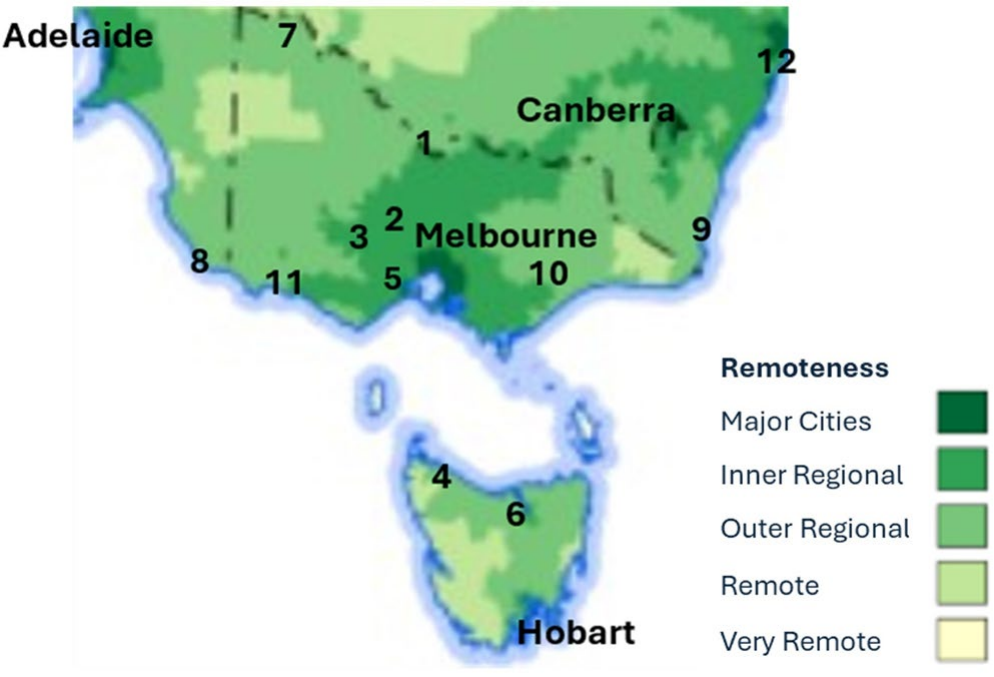
Geographic distribution of ASPREE major recruitment sites.^14^, ^15^ Major cities are Melbourne VIC, Adelaide SA, Canberra ACT, and Hobart TAS. Regional sites are (1) Albury/Wodonga, VIC/NSW; (2) Ballarat, VIC; (3) Bendigo, VIC; (4) Burnie, TAS; (5) Geelong, VIC; (6) Launceston, TAS; (7) Mildura, VIC; (8) Mount Gambier, SA; (9) Sapphire Coast, NSW; (10) Traralgon, VIC; (11) Warrnambool, VIC; (12) Wollongong, NSW. ACT, Australian Capital Territory; NSW, New South Wales; SA, South Australia; TAS, Tasmania; VIC, Victoria.

Rurality status was derived from participants' residential postcode at recruitment (Figure 1)^14^ according to the Australian Statistical Geography Standard (ASGS) Remoteness Structure: major cities, inner regional, outer regional, remote and very remote.^15^ The term ‘rural and remote’ is used for all areas outside major cities.

### 2.3 Outcome measures

The primary endpoint for ASPREE/‐XT, and investigated in this study, was DFS, and individually its components of death from any cause, incident dementia and persistent physical disability.^16^ Secondary outcomes were incident CVD events, cancer and death by underlying cause.

All‐cause mortality was confirmed by two independent sources.^16^ Dementia was assessed according to the Diagnostic and Statistical Manual of Mental Disorders, Fourth Edition (DSM‐IV) criteria.^13^, ^17^ Persistent physical disability was considered the onset of ‘a lot of difficulty’ or ‘inability’ to independently undertake any one of the six Katz activities of daily living and persisting for at least 6 months.^16^ Table S1 further details primary outcome measures.

Incident CVD events were defined as fatal coronary heart disease, non‐fatal myocardial infarction (MI), fatal or non‐fatal stroke and hospitalisation for heart failure.^16^ Cancer incidence excluded non‐melanoma skin cancers and local recurrence of the same type for individuals with a cancer history.^16^

Death by underlying cause was categorised as (a) cancer‐related; (b) fatal CVD events (MI, other coronary heart disease, sudden cardiac death, cardiac failure or stroke); (c) haemorrhagic events (fatal clinically significant bleeding and haemorrhagic stroke); and (d) all other deaths including dementia, respiratory illnesses, trauma and suicide.^16^

All incident outcomes were adjudicated using a standardised protocol by expert committees.^16^, ^17^

### 2.4 Statistical analyses

Events captured and adjudicated up to the ASPREE‐XT fourth annual visit completed by 2022 were included. Covariate data were >99.5% complete.

Adjusted Cox proportional hazards regression models were used to compare the incidence of health outcomes across three rurality status groups: major cities (referent), inner regional, and combined outer regional and remote (due to low numbers). Crude results were first adjusted for age category, sex, education and the Australian Socioeconomic Indexes for Areas—Index for Relative Socioeconomic Advantage and Disadvantage (IRSAD; Model 1); and second, for the addition of smoking status, alcohol consumption, living situation (alone or with others), body mass index, diabetes, dyslipidaemia, hypertension and depressive symptoms (Model 2). All fully adjusted models met proportional hazards assumptions (scaled Schoenfeld residuals *p* > .05).

Potential effect modification of the relationship between rurality and health outcomes by age and sex was tested by inclusion of multiplicative interaction terms and stratified accordingly. Hazard ratios by rurality status were determined for underlying causes of death.

Analyses were performed using Stata software, version 17 (StataCorpLP, College Station, TX, USA). Statistical significance was set as *p* < .05, two‐sided.

The ASPREE trial and ASPREE‐XT comply with the Declaration of Helsinki and have approval from multiple human research ethics committees.^13^, ^16^ This study was approved by the Monash Human Research Ethics Committee (#26226). All ASPREE/XT participants signed informed consent for participation.

## 3 RESULTS

### 3.1 Participant baseline characteristics by rurality status

#### 3.1.1 Demographics

Of the total Australian ASPREE cohort, rurality status was determined for 16,697 (100%). Over half lived in major cities (*n* = 8731; 52%), 36% in inner regional areas (*n* = 6002) and 12% in outer regional (*n* = 1961) and remote areas 0% (*n* = 3). There were no participants from very remote areas. Age, sex and living situation were similar by rurality group, but years of education significantly decreased with remoteness, with 58% of major city participants completing 12 years or more of education compared to 39% in outer/remote areas (Table 1). Remoteness level and socio‐economic disadvantage overlapped considerably, with 75% of those in outer regional/remote areas in the very low or low IRSAD quintiles, compared to 49% and 14% for inner regional and major cities, respectively.

**TABLE 1:** Participant baseline characteristics according to rurality status.

	Major cities (*n* = 8731)	Inner regional (*n* = 6002)	Outer regional and remote (*n* = 1964)	*p*
Age, year, *n* (%)				
70–74	5056 (58)	3457 (58)	1156 (59)	
75–84	3315 (38)	2329 (39)	744 (38)	.23
85+	360 (4)	216 (4)	64 (3)	
Sex, female, *n* (%)	4805 (55)	3281 (55)	1090 (56)	.79
Education, years, *n* (%)				
<12	3703 (42)	3493 (58)	1200 (61)	
12–15	2499 (29)	1437 (24)	442 (23)	<.001
16+	2529 (29)	1071 (18)	322 (16)	
IRSAD quintile, *n* (%)				
Very low	508 (6)	1371 (23)	869 (45)	
Low	671 (8)	1579 (26)	590 (30)	
Middle	1047 (12)	1780 (30)	335 (17)	<.001
High	2105 (24)	860 (14)	148 (8)	
Very high	4398 (50)	386 (7)	8 (0)	
Lives alone, *n* (%)	2802 (32)	1922 (32)	607 (31)	.58
Current or past smoker, *n* (%)	3836 (44)	2700 (45)	875 (45)	.44
Current or past alcohol consumption, *n* (%)	7009 (80)	4626 (77)	1542 (79)	<.001
High‐risk alcohol consumption, *n* (%)	815 (9)	531 (9)	196 (10)	<.001
Walk time no stopping, *n* (%)				
<10 min	498 (6)	402 (7)	155 (8)	
10–30 min	2609 (30)	1933 (32)	581 (30)	<.001
>30 min	5610 (64)	3654 (61)	1222 (62)	
Obese, ≥30 kg/m^2^, *n* (%)	2413 (28)	1760 (29)	592 (30)	.002
Diabetes, *n* (%)	824 (9)	608 (10)	211 (11)	.14
Hypertension, *n* (%)	6476 (74)	4565 (76)	1479 (75)	.03
Dyslipidaemia, *n* (%)	5825 (67)	4080 (68)	1401 (71)	<.001
Depressive symptoms, *n* (%)	905 (10)	551 (9)	143 (7)	<.001
Aspirin, *n* (%)	4351 (50)	2991 (50)	976 (50)	.99

*Note*: All *p*‐values are from *χ* ^2^ tests; IRSAD is the Australian Socioeconomic Indexes for Areas—Index for Relative Socioeconomic Advantage and Disadvantage, derived from residential postcode; High risk alcohol consumption was defined as >10 standard drinks per week or >4 standard drinks per occasion; Walk time no stopping is a physical activity proxy based on longest amount of walk‐time outside of home without rest in the past 2 weeks; body mass index (BMI) was measured as body weight (kg)/height (m) squared with obese ≥30 kg/m^2^, overweight 25–29.9 kg/m^2^, normal 20–24.9 kg/m^2^, underweight <20 kg/m^2^ ^16^; Diabetes was based on self‐report, fasting glucose ≥126 mg/dL or treatment for diabetes^16^; Hypertension was determined by the average of three blood pressure measurements with systolic blood pressure ≥140 mmHg or diastolic blood pressure ≥90 mmHg and/or whether the participants were on treatment for high blood pressure^16^; Dyslipidaemia was based on cholesterol‐lowering medications or low‐density lipoprotein, LDL >160 mg/dL/>4.1 mmol/L or serum cholesterol ≥212 mg/dL/≥5.5 mmol/L^16^; Depressive symptoms was based on a score of ≥8/30 on the Center for Epidemiological Studies Depression 10 scale, CES‐D‐10.^16^ Trial treatment was 100 mg per day of enteric‐coated aspirin or placebo.^16^

#### 3.1.2 Health risk factors and clinical morbidity

The proportions of current or past smokers were similar across regions (Table 1). Residents of major cities were more likely to drink alcohol than those in rural and remote areas, whereas those in outer regional/remote areas consumed alcohol at riskier levels compared to metropolitan and inner regional residents. Obesity, diabetes and dyslipidaemia were increasingly common with the degree of remoteness. Hypertension prevalence was highest among inner regional residents, and walk‐time beyond 30 min was lowest in this group. Depressive symptoms were rated highest among metropolitan residents and lowest among those living in outer regional and remote areas. There was no difference by rurality in aspirin treatment versus placebo.

### 3.2 Incident health outcomes by rurality status

#### 3.2.1 Disability‐free survival (composite primary endpoint)

**FIGURE 2:**
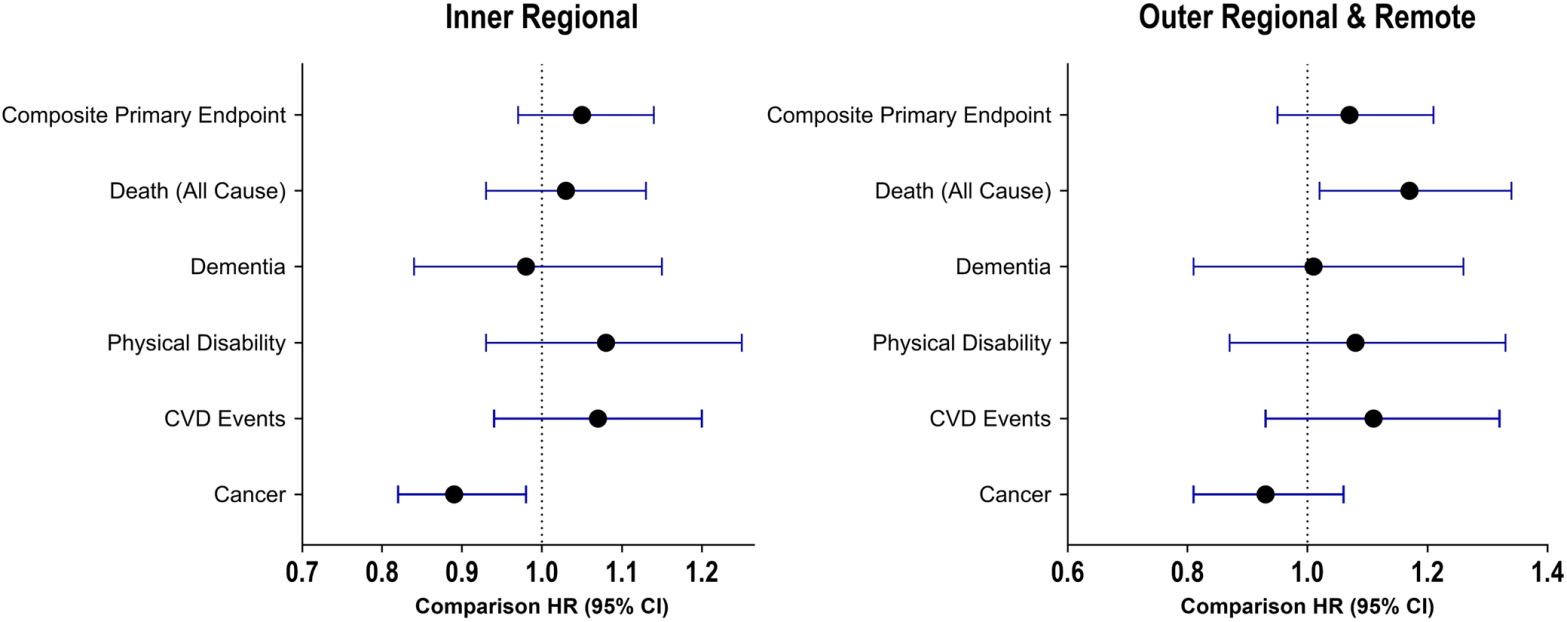
Forest plots of incident health outcome hazard ratios (HR) compared to major cities.

Over a median follow‐up of 8.3 years (interquartile range [IQR] 7.1–9.5 years), loss of DFS occurred in 22% of metropolitan residents, 24% of inner regional participants and 24% of participants in outer regional/remote areas. After covariate adjustment, no significant difference in DFS was observed across levels of residential remoteness (inner regional HR: 1.05, *p* = .27, 95% CI .97, 1.14; outer/remote HR: 1.07, *p* = .25, 95% CI .95, 1.21; Table 2 and Figure 2).

**TABLE 2:** Crude and adjusted associations between rurality and incident health outcomes.

	Major cities [ref]		Inner regional					Outer and remote				
	Events (*n*)	Rate	Events (*n*)	Rate	HR	*p*	95% CI	Events (*n*)	Rate	HR	*p*	95% CI
Composite primary endpoint (death, disability, dementia)												
Crude	1905	27.1	1445	30.3	1.13	.001	1.05, 1.20	470	30.4	1.13	.02	1.02, 1.25
Model 1					1.01	.74	.93, 1.10			1.01	.93	.89, 1.13
Model 2					1.05	.27	.97, 1.14			1.07	.25	.95, 1.21
Death (all cause)												
Crude	1358	18	1012	19.7	1.10	.02	1.02, 1.20	364	22	1.24	<.001	1.10, 1.39
Model 1					.99	.79	.89, 1.09			1.09	.20	.95, 1.25
Model 2					1.03	.58	.93, 1.13			1.17	.03	1.02, 1.34
Dementia												
Crude	578	8.25	394	8.24	1.01	.93	.89, 1.14	128	8.25	1.00	.97	.83, 1.21
Model 1					.98	.76	.84, 1.14			.98	.86	.78, 1.23
Model 2					.98	.81	.84, 1.15			1.01	.93	.81, 1.26
Physical disability												
Crude	580	8.26	459	9.62	1.17	.01	1.04, 1.33	144	9.28	1.13	.19	.94, 1.36
Model 1					1.03	.65	.89, 1.20			.99	.95	.80, 1.23
Model 2					1.08	.31	.93, 1.25			1.08	.50	.87, 1.33
CVD events												
Crude	867	12.5	659	14	1.12	.02	1.02, 1.24	220	14.4	1.16	.05	.99, 1.34
Model 1					1.05	.46	.93, 1.18			1.07	.41	.90, 1.28
Model 2					1.07	.31	.94, 1.20			1.11	.23	.93, 1.32
Cancer												
Crude	1719	26.1	1100	24.2	.93	.045	.86, .99	371	25	.96	.46	.86, 1.07
Model 1					.88	.004	.80, .96			.91	.13	.80, 1.03
Model 2					.89	.02	.82, .98			.93	.25	.81, 1.06

*Note*: Model 1 is minimally adjusted for age category, sex, education, and IRSAD; Model 2 is adjusted for model 1 plus smoking status, alcohol consumption, body mass index, living situation, diabetes, dyslipidaemia, hypertension and depressive symptoms. IRSAD is the Australian Socioeconomic Indexes for Areas—Index for Relative Socioeconomic Advantage and Disadvantage. Rate indicates crude incidence rate per 1000 person‐years. Ref indicates that major cities is the reference category with hazard ratio (HR) = 1.

#### 3.2.2 Death (all cause)

Death occurred in 16% of major city participants, 17% of participants in inner regions, and 19% of individuals living in outer/remote areas. Median follow‐up was 8.7 years (IQR 7.5–10.1 years). After covariate adjustment, a 17% higher risk of death from any cause remained for those residing in outer/remote areas (*p* = .03, 95% CI 1.02, 1.34; Table 2 and Figure 2).

#### 3.2.3 Dementia

Across a median follow‐up of 8.4 years (IQR 7.2–9.5 years), dementia was incident in 7% of participants from major cities and inner regions, and 7% of individuals living in outer/remote regions. There was no significant difference in incident dementia for inner regional or outer/remote participants when compared to those living in major cities (HR: .98, *p* = .81, 95% CI .84, 1.15; HR: 1.01, *p* = .93, 95% CI .81, 1.26; Table 2 and Figure 2).

#### 3.2.4 Persistent physical disability

Persistent physical disability occurred in 7%, 8% and 7% of individuals living in major cities, inner regions and outer/remote regions respectively, across a median follow‐up of 8.4 years (IQR 7.2–9.5 years). Compared to major cities, differences were not significant after adjustment for confounders for either inner regions (HR: 1.08, *p* = .31, 95% CI .93, 1.25) or outer/remote regions (HR: 1.08, *p* = .50, 95% CI .87, 1.33; Table 2 and Figure 2).

#### 3.2.5 Incident cardiovascular events

Cardiovascular disease events occurred in 10% of major city residents, 11% of inner regional participants and 11% of individuals living in outer/remote areas over a median of 8.4 follow‐up years (IQR 7.2–9.5 years). No significant differences compared to major cities remained after adjustment for confounders (inner regional: HR: 1.07, *p* = .31, 95% CI .94, 1.20; outer/remote: HR: 1.11, *p* = .23, 95% CI .93, 1.32; Table 2 and Figure 2).

#### 3.2.6 Cancer

Incident cancer occurred in20% of individuals living in major cities, 18% of inner regional residents and 19% of outer/remote region participants over a median follow‐up of 8.3 years (IQR 6.4–9.4 years). A lower risk (11%) of cancer incidence remained among inner regional participants compared to those in major cities (*p* = .02, 95% CI .82, .98; Table 2 and Figure 2) after full adjustment for confounders. Cancer incidence for outer/remote regions did not differ from major cities (HR: .93, *p* = .25, 95% CI .81, 1.06).

#### 3.2.7 Effect modification

For all‐cause mortality, effect modification by age was evident, with inclusion of the interaction term significant at *p* = .04. In stratified analysis, those in the 75–84 age group were at a significantly greater risk of death in outer regional and remote areas compared to major cities (HR: 1.42, *p* < .001, 95% CI 1.18, 1.70). No modification by age category or sex was observed for DFS, incident dementia, physical disability or incident CVD. For cancer, neither interaction of age nor sex was significant when included in the regression model (age category: *p* = .21; sex: *p* = .08), stratified analysis showed some indication of lower incidence among those aged under 75 years and among women (Tables S2 and S3).

#### 3.2.8 Death by underlying cause

**FIGURE 3:**
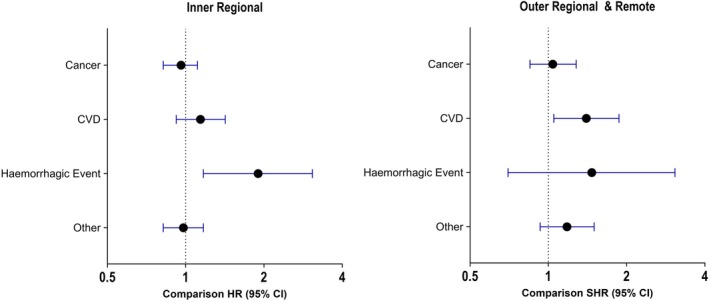
Forest plots of cause‐specific hazard ratios (HR) by underlying cause of death compared to major cities. Other deaths include dementia, respiratory illnesses, trauma and suicide.

For death by underlying cause (cancer, CVD, haemorrhagic events and all other) those living in outer/remote regions had a 40% higher risk of dying from CVD compared to those living in major cities (*p* = .02, 95% CI 1.05, 1.87). Additionally, the risk of death from haemorrhagic events was close to double among inner regional residents compared to major cities (HR: 1.90, *p* = .009, 95% CI 1.17, 3.07; Figure 3 and Table S4).

## 4 DISCUSSION

Among our cohort of 16,697 community‐dwelling older adults, small but significant differences in sociodemographic, health and behavioural factors were observed across remoteness levels, but rurality was not associated with the incidence of DFS, dementia, physical disability nor CVD events. Notably, outer regional and remote residents had an increased risk of all‐cause mortality and death from CVD events, while inner regional residents had double the risk of dying from a haemorrhagic event and a lower incidence of cancer compared to those living in major cities.

### 4.1 Risk factor prevalence by rurality

Consistent with existing literature on the rural–urban health divide, increasing remoteness was associated with fewer years of education, socioeconomic disadvantage, and higher prevalence of obesity, diabetes, high cholesterol and risky alcohol consumption.^6^, ^18^ However, inner regional participants had the lowest physical activity and highest hypertension rates. This aligns with recent research reporting underdiagnosis and suboptimal treatment of high blood pressure in some inner regions within our study area.^19^ Additionally, and contrary to national statistics,^18^ we found no difference in smoking behaviour, possibly due to a generally ‘healthier’ cohort in the ASPREE trial.^14^ Finally, although prior research tends to support heightened risk of depression among rural and remote living older adults,^20^ depressive symptoms were lower with increasing remoteness. A stronger influence of protective social and environmental aspects among this demographic has been suggested^21^ and is supported by earlier ASPREE research which found that the mental component of health‐related quality of life was also rated slightly higher with increasing rurality.^22^

### 4.2 Incident health outcomes by rurality

Our study is one of the first large longitudinal studies to investigate whether DFS, a measure of independent daily living in older adults, may vary by rurality status.^16^ However, survival free of dementia and physical disability was not found to be associated with rurality after accounting for socioeconomic and health risk factors. Similarly, incidence of dementia did not vary according to rurality, with both crude and adjusted results indicating no difference. This finding departs from prior research suggesting that individuals living in rural and remote regions may be at heightened risk of dementia.^7^, ^8^, ^9^, ^10^, ^12^ However these findings have largely been from prevalence studies, with few incidence studies undertaken to date.^8^, ^12^ Comparatively, a recent longitudinal study of 261,669 Australians, utilising data from the Sax Institute's ‘45 and up’ cohort reported a lower risk of Alzheimer's disease outside urban areas.^12^ Case ascertainment was via data‐linkage, and therefore, study results were potentially limited by misclassification bias and differences in timeliness of diagnosis.^12^ Our study thus makes an important contribution to this literature, with rigorous case ascertainment of dementia incidence and standardised adjudication processes. Likewise, we found no significant difference in persistent physical disability by remoteness.

Heightened risk of all‐cause mortality, however, was observed for those in outer regional and remote areas after adjusting for socio‐economic and health risks, specifically among those aged 75–84 years. This aligns with prior literature highlighting comparative inequity in rural–urban mortality rates.^5^, ^18^, ^23^ Drivers of this disparity may be complex; however, continuity and proximity of primary and specialist care are likely key influences.^23^, ^24^ Access to medical practitioners in Australia is around 30% lower outside major cities, with severe and persistent shortages in specialist services including cardiology, diagnostic radiology and general surgery.^25^ Further, our finding of a 40% higher risk of fatal CVD and double the risk of death from haemorrhagic events in outer/remote and inner regions, respectively, is consistent with research indicating suboptimal management of vascular risk and post‐event care within these regions.^19^, ^23^ For example, a recent 14‐year retrospective study in the Hunter New England region of Australia found CVD patients from rural or remote areas had significantly higher odds of 30‐day readmission and a higher mortality rate postacute coronary syndrome.^23^ Geospatial mapping has also indicated that older adults are overrepresented in areas further than an hour's drive from hospitals with acute cardiac facilities.^26^ Thus, with time to treatment critical, geographic distance and acute symptomology awareness may also play a considerable role,^5^ and are consistent with our finding of no significant difference by rurality for all CVD events (fatal and non‐fatal combined).

A significant and somewhat surprising finding was a lower incidence of cancer among inner regional participants compared to major cities. Given no significant difference in cancer mortality risk and past ASPREE research indicating consistent treatment across regions,^27^ this may suggest lower screening rates, delay in, or reluctance to seek diagnosis. A lower screening rate would be somewhat in line with a recent systematic review that indicated that for high‐income countries, populations outside urban areas have the lowest uptake of breast, cervical and colorectal screening.^28^ Additionally, recent national surveys have cited overlap of areas of higher disadvantage and lower screening levels.^29^ However, some Australian research suggests that inner regions may have higher screening rates than cities.^30^ Therefore, an alternative explanation may be that preventive action has been taken at an earlier age within this inner regional cohort and not reflected in mortality rates. Thus, understanding the relative contribution to incidence and mortality by rurality from cancer types with routine screening or informed testing (e.g. breast and prostate) compared to no current screening (e.g. lung), and by stage at diagnosis, may provide valuable insight.

### 4.3 Strengths and limitations

Strengths of this study include a large longitudinal cohort with considerable geographic diversity and expert committee case adjudication for incident health outcomes. Further, availability of extensive health and clinical risk factor data allowed for adjusted associations between health outcomes and rurality to be assessed. However, there are some study limitations. First, our cohort included generally ‘healthier’ older adults, hence rurality‐specific factors, protective or detrimental, may potentially not yet be apparent.^24^ Additionally, participants were not recruited from Western Australia, Queensland and the Northern Territory where distance to health services may be more extreme, and there were only three participants from remote regions and none from very remote areas. These elements may limit generalisability. Furthermore, rurality was determined at baseline, therefore, time spent in the location of residence is unknown. Finally, residual confounding is possible from factors with proxy measures (e.g. physical activity and individual level social support) and factors for which data was unavailable (e.g. air pollution and diet).

## 5 CONCLUSIONS

This study was one of the first large longitudinal studies to consider rurality in the context of independent living free of dementia and physical disability. Sociodemographic, health, and behavioural factors generally declined with increasing rurality, except for depression. However, over a median follow‐up of 8.3 years, there was no evidence that disability‐free survival, incident dementia and persistent physical disability were associated with rurality status beyond the influence of these factors. Heightened risk of all‐cause mortality, fatal CVD and haemorrhagic events outside major cities highlight the urgent need to address underlying causes, including health‐care provision and access. Additionally, further research is needed on factors contributing to lower cancer incidence in inner regions, which may require targeted health education. Overall, our study underscores the importance of high‐quality longitudinal assessment of health outcomes by rurality to drive place‐based policies and interventions to achieve healthy ageing equity.

## FUNDING INFORMATION

This work was supported by the National Institute on Aging and the National Cancer Institute at the National Institutes of Health (U01AG029824 and U19AG062682); the National Health and Medical Research Council (NHMRC) of Australia (334047 and 1127060); Monash University (Australia) and the Victorian Cancer Agency (Australia). Joanne Ryan is funded by a National Health and Medical Research Council Leadership 1 Investigator Grant (2016438). The funding bodies were not involved in study design; collection, analysis and interpretation of the data; the writing of the manuscript; or in the decision to submit the article for publication.

## CONFLICT OF INTEREST STATEMENT

No conflicts of interest declared.

## Supporting information


Appendix S1

## Data Availability

Data from the ASPREE study are available upon reasonable request. Please refer to the study website https://ams.aspree.org. All authors had full access to all of the data in the study, including statistical reports and tables related to the study.
