# Supplementary material for: Incidence of major health events across metropolitan and regional areas: A 10+ year prospective study of 16,697 older Australians
Source: Australas J Ageing. 2025 May 7;44(2):e70036. doi: 10.1111/ajag.70036 (PMC12057803; doi:10.1111/ajag.70036)
Supplement: Supplementary file 1 — Appendix S1 [file AJAG-44-0-s001.docx]

**Supplementary material**

**Death:** All-cause mortality was confirmed by two independent sources: death certificate or post-mortem report or in the absence of these, hospital or GP records, National Death Index searches and the Australian Ryerson Index online database.^1^

**Dementia:** Routine cognitive assessments (Table S1) were made at baseline, year 1 and then biennially for ASPREE, and annually during ASPREE-XT. Further evaluations (Table S1) were triggered for suspected dementia at any time for:^2,3^

- A 3MS score <78/100
- A drop of more than 10.15 points from predicted 3MS score relative to baseline (age and education adjusted)
- Memory or other cognitive concerns reported to a specialist or noted in medical records
- A clinician diagnosis of dementia
- A prescription for cholinesterase inhibitors

Additional assessments were conducted at least six weeks after the initial trigger to discount delirium.^3^ Dementia diagnosis was then adjudicated by an expert committee on DSM-IV criteria, based on evaluation outcomes and clinical case notes.^2,3^

**Table S1:** Routine and additional neurocognitive assessments

| **Timing** | **Evaluation** |
| --- | --- |
| **Routine** | Modified Mini-Mental State Examination (3MS)^4^ |
|  | Hopkins Verbal Learning Test - Revised (HVLT-R)^5^ |
|  | Controlled Oral Word Association Test (COWAT letter F)^6^ |
|  | Symbol Digit Modalities Test (SDMT)^7^ |
| **Dementia ‘Trigger’** | Alzheimer’s Disease Assessment Scale-Cognitive^8^ |
|  | Alzheimer Disease Cooperative Study Activities of Daily Living scale^9^ |
|  | Color Trails Test^7^ |
|  | Lurian Overlapping Figures^10^ |
|  | Brain CT or MRI and dementia-panel blood tests |

**Physical Disability:** Assessed by the participant’s self-reported ability to perform 6 basic Activities of Daily Living (ADLs), including eating, bathing, dressing, walking, transferral (from bed or chair) and toileting; based on the original Katz ADLs.^1^ Response options were 1) no difficulty, (2) a little difficulty, (3) some difficulty, (4) a lot of difficulty, (5) unable to perform and (6) requires assistance to perform. Assessments were made at baseline and via six-monthly phone calls and annual visits thereafter. The endpoint was a response of either ‘a lot of difficulty’ or ‘unable to perform’ or ‘requiring assistance with’ that persisted for six months.^1^

**Table S2:** Age stratification of fully adjusted associations between rurality and incident health outcomes compared to major cities

|  |  | **<75 Years** | | | | | | |  | **75 to 84 Years** | | | | | | |  | **≥ 85 Years** | | | | | | |
| --- | --- | --- | --- | --- | --- | --- | --- | --- | --- | --- | --- | --- | --- | --- | --- | --- | --- | --- | --- | --- | --- | --- | --- | --- |
|  |  | **Inner Regional** | | |  | **Outer & Remote** | | |  | **Inner Regional** | | |  | **Outer & Remote** | | |  | **Inner Regional** | | |  | **Outer & Remote** | | |
|  |  | **HR** | ***p*** | **95% CI** |  | **HR** | ***p*** | **95% CI** |  | **HR** | ***p*** | **95% CI** |  | **HR** | ***p*** | **95% CI** |  | **HR** | ***p*** | **95% CI** |  | **HR** | ***p*** | **95% CI** |
|  |  |  |  |  |  |  |  |  |  |  |  |  |  |  |  |  |  |  |  |  |  |  |  |  |
| **Composite Primary Endpoint** |  | 1.01 | 0.91 | .88, 1.15 |  | .93 | 0.44 | .76, 1.12 |  | 1.08 | 0.21 | .96, 1.21 |  | 1.17 | 0.06 | 1.00, 1.37 |  | .97 | 0.81 | .74, 1.27 |  | 1.15 | 0.48 | .78, 1.72 |
|  |  |  |  |  |  |  |  |  |  |  |  |  |  |  |  |  |  |  |  |  |  |  |  |  |
| **Death** |  | .99 | 0.88 | .84, 1.17 |  | .89 | 0.33 | .70, 1.13 |  | 1.09 | 0.21 | .95, 1.25 |  | 1.42 | <0.001 | 1.18, 1.70 |  | .83 | 0.20 | .62, 1.10 |  | 1.08 | 0.71 | .71, 1.64 |
|  |  |  |  |  |  |  |  |  |  |  |  |  |  |  |  |  |  |  |  |  |  |  |  |  |
| **Dementia** |  | 1.08 | 0.54 | .84, 1.40 |  | 1.16 | 0.40 | .82, 1.66 |  | .93 | 0.48 | .75, 1.14 |  | .96 | 0.80 | .71, 1.30 |  | .84 | 0.58 | .46, 1.56 |  | .62 | 0.34 | .23, 1.66 |
|  |  |  |  |  |  |  |  |  |  |  |  |  |  |  |  |  |  |  |  |  |  |  |  |  |
| **Persistent Disability** |  | .93 | 0.57 | .73, 1.19 |  | .89 | 0.53 | .63, 1.27 |  | 1.12 | 0.27 | .92, 1.38 |  | 1.10 | 0.50 | .83, 1.47 |  | 1.34 | 0.21 | .85, 2.11 |  | 1.73 | 0.12 | .86, 3.46 |
|  |  |  |  |  |  |  |  |  |  |  |  |  |  |  |  |  |  |  |  |  |  |  |  |  |
| **CVD** |  | 1.05 | 0.58 | .87, 1.27 |  | 1.01 | 0.97 | .77, 1.31 |  | 1.07 | 0.44 | .90, 1.27 |  | 1.20 | 0.15 | .94, 1.53 |  | .97 | 0.89 | .63, 1.50 |  | 1.13 | 0.72 | .58, 2.18 |
|  |  |  |  |  |  |  |  |  |  |  |  |  |  |  |  |  |  |  |  |  |  |  |  |  |
| **Cancer** |  | .82 | 0.001 | .72, .92 |  | .77 | 0.004 | .64, .92 |  | .99 | 0.94 | .86, 1.14 |  | 1.14 | 0.21 | .93, 1.38 |  | 1.05 | 0.83 | .67, 1.65 |  | 1.29 | 0.44 | .68, 2.43 |
|  |  |  |  |  |  |  |  |  |  |  |  |  |  |  |  |  |  |  |  |  |  |  |  |  |

**Table S3:** Sex stratification of fully adjusted associations between rurality and incident health outcomes compared to major cities

|  |  | **Male** | | | | | | |  | **Female** | | | | | | |
| --- | --- | --- | --- | --- | --- | --- | --- | --- | --- | --- | --- | --- | --- | --- | --- | --- |
|  |  | **Inner Regional** | | |  | **Outer & Remote** | | |  | **Inner Regional** | | |  | **Outer & Remote** | | |
|  |  | **HR** | ***p*** | **95% CI** |  | **HR** | ***p*** | **95% CI** |  | **HR** | ***p*** | **95% CI** |  | **HR** | ***p*** | **95% CI** |
|  |  |  |  |  |  |  |  |  |  |  |  |  |  |  |  |  |
| **Composite Primary Endpoint** | | 1.04 | 0.49 | .93, 1.17 |  | 1.08 | 0.35 | .92, 1.28 |  | 1.05 | 0.41 | .94, 1.18 |  | 1.06 | 0.52 | .90, 1.24 |
|  |  |  |  |  |  |  |  |  |  |  |  |  |  |  |  |  |
| **Death** |  | 1.05 | 0.48 | .92, 1.20 |  | 1.10 | 0.31 | .91, 1.33 |  | 1.00 | 0.97 | .87, 1.16 |  | 1.23 | 0.04 | 1.01, 1.50 |
|  |  |  |  |  |  |  |  |  |  |  |  |  |  |  |  |  |
| **Dementia** |  | .92 | 0.45 | .73, 1.15 |  | 1.12 | 0.49 | .81, 1.54 |  | 1.03 | 0.77 | .83, 1.28 |  | .92 | 0.61 | .67, 1.26 |
|  |  |  |  |  |  |  |  |  |  |  |  |  |  |  |  |  |
| **Persistent Disability** |  | 1.15 | 0.23 | .91, 1.46 |  | 1.20 | 0.28 | .86, 1.68 |  | 1.02 | 0.86 | .84, 1.23 |  | .97 | 0.82 | .74, 1.28 |
|  |  |  |  |  |  |  |  |  |  |  |  |  |  |  |  |  |
| **CVD** |  | .97 | 0.74 | .82, 1.15 |  | .99 | 0.93 | .78, 1.26 |  | 1.18 | 0.07 | .99, 1.42 |  | 1.27 | 0.07 | .98, 1.63 |
|  |  |  |  |  |  |  |  |  |  |  |  |  |  |  |  |  |
| **Cancer** |  | .93 | 0.20 | .82, 1.04 |  | .86 | 0.10 | .72, 1.03 |  | .85 | 0.02 | .74, .98 |  | 1.00 | 0.96 | .83, 1.21 |
|  |  |  |  |  |  |  |  |  |  |  |  |  |  |  |  |  |

**Table S4:** Crude and adjusted associations between rurality and underlying cause of death

|  | **Major Cities [Ref]** | |  | **Inner Regional** | | | |  | **Outer & Remote** | | | |
| --- | --- | --- | --- | --- | --- | --- | --- | --- | --- | --- | --- | --- |
|  | **Events (n)** | **HR** |  | **Events (n)** | **HR** | ***p-*value** | **95% CI** |  | **Events (n)** | **HR** | ***p-*value** | **95% CI** |
| **Cancer** |  |  |  |  |  |  |  |  |  |  |  |  |
| *Crude* | 582 | 1 |  | 427 | 1.08 | 0.21 | .96, 1.23 |  | 151 | 1.20 | 0.05 | 1.00, 1.43 |
| *Model 1* |  |  |  |  | .92 | 0.26 | .79, 1.07 |  |  | .98 | 0.87 | .80, 1.21 |
| *Model 2* |  |  |  |  | .96 | 0.56 | .82, 1.11 |  |  | 1.04 | 0.69 | .85, 1.28 |
|  |  |  |  |  |  |  |  |  |  |  |  |  |
| **CVD** |  |  |  |  |  |  |  |  |  |  |  |  |
| *Crude* | 271 | 1 |  | 224 | 1.23 | 0.02 | 1.03, 1.47 |  | 88 | 1.50 | 0.001 | 1.18, 1.91 |
| *Model 1* |  |  |  |  | 1.10 | 0.40 | .88, 1.36 |  |  | 1.30 | 0.07 | .98, 1.74 |
| *Model 2* |  |  |  |  | 1.14 | 0.22 | .92, 1.42 |  |  | 1.40 | 0.02 | 1.05, 1.87 |
|  |  |  |  |  |  |  |  |  |  |  |  |  |
| **Haemorrhagic Event** |  |  |  |  |  |  |  |  |  |  |  |  |
| *Crude* | 54 | 1 |  | 55 | 1.51 | 0.03 | 1.04, 2.20 |  | 12 | 1.02 | 0.94 | .55, 1.91 |
| *Model 1* |  |  |  |  | 1.90 | 0.008 | 1.18, 3.06 |  |  | 1.44 | 0.33 | .69, 2.99 |
| *Model 2* |  |  |  |  | 1.90 | 0.009 | 1.17, 3.07 |  |  | 1.47 | 0.31 | .70, 3.07 |
|  |  |  |  |  |  |  |  |  |  |  |  |  |
| **Other** |  |  |  |  |  |  |  |  |  |  |  |  |
| *Crude* | 451 | 1 |  | 306 | 1.01 | 0.91 | (0.87, 1.17) |  | 113 | 1.16 | 0.15 | .95, 1.43 |
| *Model 1* |  |  |  |  | .93 | 0.42 | (0.78, 1.11) |  |  | 1.08 | 0.51 | .85, 1.38 |
| *Model 2* |  |  |  |  | .98 | 0.82 | (0.82, 1.17) |  |  | 1.18 | 0.18 | .93, 1.50 |

Model 1 is minimally adjusted for age category, sex, education and IRSAD. Model 2 is adjusted for Model 1 with the addition of smoking status, alcohol consumption, BMI, diabetes, dyslipidaemia, hypertension, depression, living situation (alone or with family/others). Other deaths include dementia, respiratory illnesses, trauma and suicide. Ref, indicates that major cities is the reference category.

**REFERENCES**

1. McNeil JJ, Nelson MR, Woods RL, et al. Effect of Aspirin on All-Cause Mortality in the Healthy Elderly. *N Engl J Med*. 2018;379(16):1519-1528. doi:10.1056/NEJMoa1803955

2. Ernst ME, Broder JC, Wolfe R, et al. Health Characteristics and Aspirin Use in Participants at the Baseline of the ASPirin in Reducing Events in the Elderly – eXTension (ASPREE-XT) Observational Study. *Contemp Clin Trials*. 2023;130:107231. doi:10.1016/j.cct.2023.107231

3. Ryan J, Storey E, Murray AM, et al. Randomized placebo-controlled trial of the effects of aspirin on dementia and cognitive decline. *Neurology*. 2020;95(3):e320-e331. doi:10.1212/wnl.0000000000009277

4. Bland RC, Newman SC. Mild dementia or cognitive impairment: The Modified Mini-Mental State Examination (3MS) as a screen for dementia. *Can J Psychiatry*. 2001;46(6):506-510. doi:10.1177/070674370104600604

5. Benedict RHB, Schretlen D, Groninger L, Brandt J. Hopkins Verbal Learning Test – Revised: Normative Data and Analysis of Inter-Form and Test-Retest Reliability. *Clin Neuropsychol*. Routledge; 1998;12(1):43-55. doi:10.1076/clin.12.1.43.1726

6. Ross TP. The reliability of cluster and switch scores for the Controlled Oral Word Association Test. *Arch Clin Neuropsychol*. 2003;18(2):153-164. doi:10.1016/S0887-6177(01)00192-5

7. Study design of ASPirin in Reducing Events in the Elderly (ASPREE): A randomized, controlled trial. *Contemp Clin Trials*. 2013;36(2):555-564. doi:10.1016/j.cct.2013.09.014

8. Kueper JK, Speechley M, Montero-Odasso M. The Alzheimer's Disease Assessment Scale-Cognitive Subscale (ADAS-Cog): Modifications and Responsiveness in Pre-Dementia Populations. A Narrative Review. *J Alzheimers Dis*. 2018;63(2):423-444. doi:10.3233/jad-170991

9. Galasko D, Bennett DA, Sano M, Marson D, Kaye J, Edland SD. ADCS Prevention Instrument Project: Assessment of Instrumental Activities of Daily Living for Community-dwelling Elderly Individuals in Dementia Prevention Clinical Trials. *Alzheimer Dis Assoc Disord*. 2006;20(SUPPL. 3):S152-S169. doi:10.1097/01.wad.0000213873.25053.2b

10. Reid W, Broe G, Creasey H, et al. Age at onset and pattern of neuropsychological impairment in mild early-stage Alzheimer disease: A study of a community-based population. *Arch Neurol*. 1996;53(10):1056-1061. doi:10.1001/archneur.1996.00550100142023
